# Knowledge and practices related to snakebite prevention, China: a cross-sectional study

**DOI:** 10.2471/BLT.23.290169

**Published:** 2024-01-31

**Authors:** Lanfen He, Chuanzhu Lv, Xingyue Song, Wenjie Hao, Juntao Wang, Yanlan Hu, Yu Chen, Yong Gan, Xiaotong Han, Shijiao Yan

**Affiliations:** a International School of Public Health and One Health, Hainan Medical University, No 3 Xueyuan Road, Longhua Zone, Haikou, Hainan Province, 571199, China.; b Emergency Medicine Center of the Sichuan Provincial People's Hospital, University of Electronic Science and Technology of China, Chengdu, China.; c Department of Emergency Medicine, the Second Affiliated Hospital of Hainan Medical University, Haikou, China.; d Department of Social Medicine and Health Management at the School of Public Health, Huazhong University of Science and Technology, Wuhan, China.; e Department of Emergency Medicine, Hunan Provincial People's Hospital of Hunan Normal University, Changsha, China.

## Abstract

**Objective:**

To assess knowledge and practices related to snakebite prevention among Chinese residents.

**Methods:**

By using a multistage random sampling approach augmented by snowball sampling, we surveyed residents from 10 provinces, one municipality and one autonomous region south of the Yangtze River Basin between May 2022 and February 2023. We supplemented the data with a national online survey. We used a *χ*^2^-test to identify differences in knowledge and behaviour across various demographic characteristics. We conducted multifactor logistic regression analyses to evaluate factors potentially influencing snakebite knowledge and practices.

**Findings:**

We obtained 55 775 valid survey responses, 16 200 respondents from the face-to-face survey and 39 575 respondents from the online survey. Only 25.7% (14 325) respondents demonstrated adequate knowledge about snakebites whereas 25.6% (14 295) respondents knew basic first-aid practices or preventive behaviours. Age, marital status, educational attainment, occupation, type of residence and frequency of exposure to nature are significant independent variables affecting snakebite knowledge (*P*-values: < 0.05). On the other hand, gender, age, marital status, educational attainment, occupation and type of residence were significant independent variables affecting the behaviour of snakebite prevention and first aid (*P*-values: < 0.05).

**Conclusion:**

There is a notable shortfall in knowledge, first aid and preventive behaviours among Chinese residents regarding snakebites. Misguided first aid practices can severely compromise the effectiveness of evidence-based therapeutic interventions. Consequently, improving health education concerning snakes and snakebites in this population is needed.

## Résumé

**Objectif:**

Évaluer les connaissances et pratiques liées à la prévention des morsures de serpent chez les personnes résidant en Chine.

**Méthodes:**

À l'aide d'une approche d'échantillonnage aléatoire à plusieurs degrés agrémentée d'un échantillonnage en boule de neige, nous avons interrogé les habitants de dix provinces, d'une municipalité et d'une région autonome au sud du bassin du fleuve Yangtsé entre mai 2022 et février 2023. Nous avons complété ces données par une enquête nationale en ligne. Nous avons utilisé le test du *χ*^2^ pour repérer les différences entre les connaissances et comportements en lien avec plusieurs caractéristiques démographiques. Enfin, nous avons effectué des analyses multifactorielles de régression logistique afin de déterminer les facteurs susceptibles d'influencer les connaissances et pratiques relatives aux morsures de serpent.

**Résultats:**

Nous avons enregistré 55 775 réponses valides dans le cadre de l'enquête: 16 200 lors d'entretiens en face-à-face et 39 575 en ligne. Seulement 25,7% (14 325) des participants étaient suffisamment informés sur les morsures de serpent, tandis que 25,6% (14 295) des participants connaissaient les premiers soins de base ou les comportements préventifs. L'âge, l'état civil, le niveau d'éducation, la profession, le type d'habitation et la fréquence d'exposition à la nature étaient autant de variables indépendantes ayant un impact non négligeable sur les connaissances en matière de morsures de serpent (valeurs-*p*: < 0,05). En outre, le genre, l'âge, l'état civil, le niveau d'éducation, la profession et le type d'habitation étaient autant de variables indépendantes ayant un impact non négligeable sur le comportement en matière de prévention des morsures de serpent et de premiers soins (valeurs-*p*: < 0,05).

**Conclusion:**

Chez les personnes résidant en Chine, les connaissances, les pratiques en matière de premiers soins et les comportements préventifs relatifs aux morsures de serpent ne sont pas suffisants. Accomplir les mauvais gestes lors des premiers soins peut sérieusement compromettre l'efficacité des interventions thérapeutiques éprouvées. Il est donc impératif de mieux former cette population aux serpents et à leurs morsures.

## Resumen

**Objetivo:**

Evaluar los conocimientos y las prácticas relacionados con la prevención de las mordeduras de serpiente entre los residentes chinos.

**Métodos:**

Mediante el uso de un enfoque de muestreo aleatorio de etapas múltiples aumentado por el muestreo de bola de nieve, se encuestó a los residentes de 10 provincias, un municipio y una región autónoma al sur de la cuenca del río Yangtze entre mayo de 2022 y febrero de 2023. Se complementaron los datos con una encuesta nacional en línea. Se aplicó una prueba χ^2^ para identificar las diferencias en los conocimientos y los comportamientos según diversas características demográficas. Además, se realizaron análisis de regresión logística multifactorial para evaluar los factores que podrían influir en los conocimientos y las prácticas relacionados con las mordeduras de serpiente.

**Resultados:**

Se obtuvieron 55 775 respuestas válidas, 16 200 de la encuesta presencial y 39 575 de la encuesta en línea. Solo el 25,7% (14 325) de los encuestados demostró tener conocimientos adecuados sobre las mordeduras de serpiente, mientras que el 25,6% (14 295) conocía prácticas básicas de primeros auxilios o conductas preventivas. La edad, el estado civil, el nivel de estudios, la ocupación, el tipo de residencia y la frecuencia de exposición a la naturaleza son variables independientes significativas que afectan a los conocimientos sobre las mordeduras de serpiente (valores de *p*: <0,05). Por otra parte, el sexo, la edad, el estado civil, el nivel de estudios, la ocupación y el tipo de residencia fueron variables independientes significativas que afectaron al comportamiento de prevención de las mordeduras de serpiente y a los primeros auxilios (valores de *p*: <0,05).

**Conclusión:**

Los residentes chinos presentan un notable déficit de conocimientos, primeros auxilios y conductas preventivas en relación con las mordeduras de serpiente. Las prácticas erróneas de primeros auxilios pueden comprometer gravemente la eficacia de las intervenciones terapéuticas basadas en la evidencia. En consecuencia, es necesario mejorar la educación sanitaria relativa a las serpientes y a sus mordeduras en esta población.

## ملخص

**الغرض:**

تقييم المعرفة والممارسات المتعلقة بالوقاية من لدغات الثعابين بين السكان في الصين.

**الطريقة:**

باستخدام نهج جمع العينات العشوائية متعدد المراحل، والمعزز بجمع عينات كرة الثلج، قمنا بمسح لآراء سكان من 10 مقاطعات، وبلدية واحدة، ومنطقة ذاتية الحكم جنوب حوض نهر اليانغتسي، في الفترة ما بين مايو/أيار 2022 وفبراير/شباط 2023. وقمنا بإكمال البيانات بالاستعانة بمسح وطني عبر الإنترنت. وقمنا باستخدام اختبار *χ2 * لتحديد الاختلافات في المعرفة والسلوك عبر الخصائص السكانية المختلفة. وأجرينا تحليلات التحوّف اللوجستي متعددة العوامل لتقييم العوامل التي يحتمل أن تؤثر على المعرفة والممارسات المتعلقة بلدغات الثعابين.

**النتائج:**

حصلنا على 55775 استجابة صالحة من المسح، و16200 مشاركًا في المسح وجهًا لوجه، و39575 مشتركًا في المسح عبر الإنترنت. أظهر %25.7 فقط (14325) من المشاركين معرفة مناسبة بخصوص لدغات الثعابين، بينما كان %25.6 (14295) من المشاركين يعرفون ممارسات الإسعافات الأولية الأساسية أو السلوكيات الوقائية. يعد كل من العمر، والحالة الاجتماعية، والدرجة التعليمية، والمهنة، ونوع المسكن، وتكرار التعرض للطبيعة، متغيرات مستقلة ملموسة تؤثر على معرفة لدغات الثعابين (قيم نسبة الاحتمال: < 0.05). وعلى الجانب الآخر، كان كل من الجنس، والعمر، والحالة الاجتماعية، والدرجة التعليمية، والمهنة، ونوع المسكن، متغيرات مستقلة ملموسة تؤثر على سلوك الوقاية من لدغات الثعابين والإسعافات الأولية (قيم نسبة الاحتمال: < 0.05).

**الاستنتاج:**

هناك نقص ملحوظ في المعرفة، والإسعافات الأولية، والسلوكيات الوقائية، بين السكان في الصين فيما يتعلق بلدغات الثعابين. يمكن أن تؤدي ممارسات الإسعافات الأولية الخاطئة إلى الإخلال بشدة بفعالية التدخلات العلاجية القائمة على الأدلة. وبالتالي، فإن هناك حاجة إلى تحسين التثقيف الصحي فيما يتعلق بالثعابين ولدغات الثعابين في هذه الفئة من السكان.

## 摘要

**目的:**

评估中国居民预防蛇咬伤的相关知识和实践。

**方法:**

2022 年 5 月至 2023 年 2 月期间,我们采用结合了滚雪球抽样的多阶段随机抽样方法,对长江流域以南 10 个省、1 个直辖市和 1 个自治区的居民进行了调查。我们通过一项全国性的在线调查对数据进行了补充。我们使用 *χ*^2^ 检验来确定不同人口统计学特征的居民在知识和行为方面的差异。我们进行了多因素逻辑回归分析,以评估可能影响蛇咬伤相关知识和实践的因素。

**结果:**

我们获得了 55,775 份有效的调查回复,其中 16,200 名受访者来自面对面调查,39,575 名受访者来自在线调查。只有 25.7%(14 325 名)受访者对蛇咬伤有足够的了解,而 25.6%(14 295 名)受访者知道基本的急救实践或预防行为。年龄、婚姻状况、受教育程度、职业、住宅类型以及接触大自然的频率是影响了解蛇咬伤知识的重要独立变量(*P* 值:< 0.05)。另一方面,性别、年龄、婚姻状况、受教育程度、职业和住宅**类型是影响**了解蛇咬伤预防和急救行为的重要独立变量(*P* 值:< 0.05)。

**结论:**

中国居民对蛇咬伤的知识、急救和预防行为的了解明显不足。误导性的急救实践会严重影响循证治疗干预措施的有效性。因此,需要在这类人群中加强有关蛇和蛇咬伤的健康教育。

## Резюме

**Цель:**

Оценить знания и практику профилактики змеиных укусов среди жителей Китая.

**Методы:**

С использованием многоступенчатой случайной выборки, дополненной выборкой по методу снежного кома, за период с мая 2022 года по февраль 2023 года был проведен опрос жителей 10 провинций, одного муниципалитета и одного автономного района к югу от бассейна реки Янцзы. Полученные данные были дополнены национальным электронным опросом мнений. Для выявления различий в знаниях и поведении по различным демографическим характеристикам использовался критерий *χ*^2^. Для оценки факторов, потенциально влияющих на знания о змеиных укусах и практику их применения, был проведен многофакторный логистический регрессионный анализ.

**Результаты:**

В ходе опроса было получено 55 775 достоверных ответов: от 16 200 респондентов в ходе очного опроса и от 39 575 респондентов в ходе электронного опроса мнений. Всего 25,7% респондентов (14 325) обладают достаточными знаниями о змеиных укусах, в то время как 25,6% респондентов (14 295) знакомы с основными методами оказания первой помощи или профилактическими моделями поведения. Возраст, семейное положение, уровень образования, профессия, место жительства и периодичность пребывания на природе являются значимыми независимыми переменными, влияющими на знания о змеиных укусах (*P*-значения: < 0,05). С другой стороны, пол, возраст, семейное положение, уровень образования, профессия и место жительства являются значимыми независимыми переменными, оказывающими влияние на поведение в отношении профилактики змеиных укусов и оказания первой помощи (*P*-значения: < 0,05).

**Вывод:**

Жители Китая сталкиваются с заметным недостатком знаний, навыков оказания первой помощи и профилактического поведения в отношении змеиных укусов. Ошибочные методы оказания первой помощи могут серьезно снизить эффективность терапевтического воздействия, основанного на доказательствах. Следовательно, необходимо повышать уровень санитарного просвещения в отношении змей и змеиных укусов среди населения.

## Introduction

Snakebite envenoming is an important public health problem in low- and middle-income tropical countries. Venomous snakebites can cause serious local and systemic symptoms, such as tissue damage, systemic muscular toxicity, systemic bleeding, acute kidney injury and death.^1^^,^^2^ The World Health Organization (WHO) recommends that snakebite be defined as a neglected environmental and occupational disease.^1^^,^^3^ Approximately 7400 people are bitten by snakes every day, resulting in 81 000 to 138 000 deaths per year and 400 000 people with permanent physical or mental disabilities.^4^^,^^5^ Research indicates that it is primarily male farmers, plantation workers and herders who are bitten in the lower limbs, with a higher prevalence of snakebites occurring in the summer and fall than winter and spring.^6^^–^^8^

In China, a large number of people working in agriculture, forestry and fisheries are at the risk of snakebite because these workers spend most of their time outdoors and are more likely to have regular interaction with snakes. In the country, there are an estimated 210 different species of snakes, with more than 60 species of venomous snakes and more than 10 species of highly venomous snakes. These highly venomous snakes are classified into: (i) neurotoxic; (ii) haematotoxic; (iii) cytotoxic; and (iv) mixed-venomous snakes.^8^ Previous studies indicate that if individuals have basic knowledge of snakebite prevention and first aid, the risk of being bitten as well as the risk of complications, disability and death may be reduced.^9^

Studies conducted in Brazil, India and Myanmar demonstrate a lack of knowledge about snakes and snakebite prevention and appropriate first-aid behaviour among the local populations. For example, in Brazil, 50.7% (67/132) of people surveyed do not give any first aid after being bitten; 49.3% (65/132) provide inappropriate first aid techniques; and 83.7% (108/129) do not use adequate preventive measures to avoid contact with the snake.^10^^–^^12^ In India, one study found that 65.0% (13/20) of schoolteachers surveyed lack basic knowledge about snakes and snakebites.^11^

We conducted a cross-sectional study to understand current knowledge and practices related to snakes and snakebite among Chinese people, especially residing in the vicinity of the southern Yangtze River Basin. The purpose of our study is to provide evidence for relevant departments in China to formulate policies and protocols for health education.

## Methods

We conducted a cross-sectional study in the vicinity of the southern Yangtze River Basin, China, from May 2022 to February 2023 using multistage random and snowball sampling to obtain our study sample. Based on a literature review and our combined work practice, we selected 10 provinces (Fujian, Guangdong, Guizhou, Hainan, Hubei, Hunan, Jiangxi, Sichuan, Yunnan and Zhejiang), one municipality (Chongqing) and one autonomous region (Guangxi) with known incidents of severe snakebites. We used the convenience sampling method to select three cities from each province, municipality and autonomous region: three districts or counties from each city, and three villages from each district or county for a total of 324 residential areas.

We conducted a survey with between 30 and 50 residents from each community or village using the chance encounter or convenience sampling method. Where literacy levels were low, we provided oral questionnaires. Literate residents self-filled the survey form and returned them to project enumerators. All surveys were distributed and collected face-to-face at the village level. At the same time, we used the instant message software WeChat (Shenzhen Tencent Computer System Co., Ltd, Shenzhen, China) and Tencent QQ (Shenzhen Tencent Computer System Co., Ltd, Shenzhen, China) to show advertisements or pop-up invitations in other regions to recruit random residents to participate in the same online survey we used in the villages. 

This study was approved by the medical ethics committee of Hainan Medical University (ethics number: HYLL-2022-226), and informed consent was obtained from each participant before completing the survey or questionnaire. The preface of the survey or questionnaire explained the purpose of the study, and all data were collected without recording any identifying data except for gender, i.e. male or female.

### Questionnaire design and measurement

We designed our survey based on a combination of literature review, expert consensus on snakebite, interviews with experts and focus group discussions. In addition, a pre-survey was conducted in three cities of Hainan province to assess the validity of the survey and make any necessary changes or improvements. We designed the survey to assess overall knowledge of snakes and snakebite prevention and first aid. The survey included topics on demographic characteristics, snakebite-related knowledge and experience, and snakebite prevention and first-aid behaviour. A translated version of the questionnaire is available in our online repository.^13^

### Quality Control

We designed our online survey using Questionnaire Star (Question Star, Hanover, Germany), which provided us with a weblink that we sent to participants using advertisements and popups to participate via QQ and WeChat. All participants were assigned a unique identifier while completing the online survey to prevent repeat submissions.

### Statistical Analysis

We collated all data into Microsoft Excel (Microsoft, Redmond, United States of America) and imported it into Questionnaire Star platform. The data were analysed using SPSS, version 25.0 (IBM, Chicago, USA). The knowledge survey consisted of 10 multiple-choice proficiency questions. Each question had four possible correct answers. Scores ranged between 0 and 4 for each question for a total possible points of 40. Each question also contained the option “I do not know” as a possible response. Choosing “I do not know” resulted in a score of 0 points. If the total score of a participant was 20 points or less, we considered the participant as having limited knowledge, and we considered a participant with a total score of  21 or above (greater than 50% correct) as having sufficient knowledge. 

The practices section consisted of five multiple-choice questions, of which one question contained only incorrect answers as options. Each question also contained the option “I do not know”, and the scoring method was the same as that of the knowledge section described above. Questions with no correct answers were counted as zero in the total score; and the total score for the behavioural component was 16 points. If the total score of a participant was  8 points or less, we considered the participant to have limited knowledge, and if the participant had a score of 9 points or more (greater than 50% correct), we considered them as having sufficient knowledge. 

We used univariate *χ*^2^-tests to evaluate variables for inclusion in multivariate analyses. Statistically significant variables in the univariate analysis were taken as independent variables, and knowledge and practices (converted into binomial data as limited knowledge: 0; sufficient knowledge: 1) were taken as dependent variables. We then used a multifactor logistic regression model to assess demographic and social factors associated with knowledge and practices related to snakebite among the participants. To do this, we used binary logistic regression where a *P*-value of < 0.05 was considered statistically significant.

## Results

A total of 56 804 individuals responded to the survey. We excluded 1029 surveys due to logical errors. Our study therefore includes 55 775 respondents: 16 200 respondents from the face-to-face survey and 39 575 respondents from the online survey. In total, there were 32 017 males (57.4%) and 23 758 females (42.6%). Most respondents were between 18 and 60 years old (84.5%; 47 119); and more than half were married (60.4%; 33 686). Of the respondents, 60.0% (33 442) had a high school or technical secondary school education or less, and farmers accounted for 15.7% (8738). Most respondents lived in cement buildings (70.1%; 39 104), and 60.1% (33 526) had high exposure risk (time spent in nature). 

Of the 6 837 respondents who reported being bitten by a snake, 68.0% (4652) were males and most were between the age of 18 and 60 years (90.3%; 6174), and had education levels at secondary or technical school or below (64.1%; 4381). Among the 591 respondents involved in land and sea field operations, 171 (28.9%) reported being bitten by a snake. Other occupations with higher percentages of snakebite incidents included snake catchers or breeders (26.1%; 219/839), service workers (16.6%; 1136/6842), farmers (15.3%; 1334/8738) and workers (14.8%; 1247/8413). People who spent most of their time in nature were at elevated risk of exposure to snakebite (82.5%; 5643/6837; Table 1).

**Table 1:** Demographic characteristics of respondents, by snakebite experience, China, 2022

Characteristic	No. (%)		
	Snakebite (*n* = 6 837)	No snakebite (*n* = 48 938)	Total (*n* = 55 775)
**Gender **			
Male	4 652 (68.0)	27 365 (55.9)	32 017 (57.4)
Female	2 185 (32.0)	21 573 (44.1)	23 758 (42.6)
**Age, years**			
< 18	433 (6.3)	2 688 (5.5)	3 121 (5.6)
18–40	5 262 (77.0)	28 035 (57.3)	33 297 (59.7)
41–60	912 (13.3)	12 910 (26.4)	13 822 (24.8)
> 60	230 (3.4)	5 305 (10.8)	5 535 (9.9)
**Marital status**			
Married	3 541 (51.8)	30 145 (61.6)	33 686 (60.4)
Single	2 606 (38.1)	14 242 (29.1)	16 848 (30.2)
Divorced	527 (7.7)	3 146 (6.4)	3 673 (6.6)
Widowed	163 (2.4)	1 405 (2.9)	1 568 (2.8)
**Education level**			
No education	603 (8.8)	3 243 (6.6)	3 846 (6.9)
Primary school	936 (13.7)	5 895 (12.0)	6 831 (12.2)
Middle school	1 333 (19.5)	8 974 (18.3)	10 307 (18.5)
High school or technical secondary school	1 509 (22.1)	10 949 (22.4)	12 458 (22.3)
Vocational institution	1 331 (19.5)	9 643 (19.7)	10 974 (19.7)
Bachelor’s degree or above	1 125 (16.5)	10 234 (20.9)	11 359 (20.4)
**Occupation**			
Farmer	1 334 (19.5)	7 404 (15.1)	8 738 (15.7)
Skilled labourer	1 247 (18.2)	7 166 (14.6)	8 413 (15.1)
Service worker	1 136 (16.6)	5 706 (11.7)	6 842 (12.3)
Self-employed	345 (5.0)	3 236 (6.6)	3 581 (6.4)
Freelance	373 (5.5)	4 339 (8.9)	4 712 (8.4)
Snake catcher or breeder	219 (3.2)	620 (1.3)	839 (1.5)
Land and sea field operator	171 (2.5)	420 (0.9)	591 (1.1)
Cadre employee	1 038 (15.2)	7 653 (15.6)	8 691 (15.6)
Medical employee	499 (7.3)	5 322 (10.9)	5 821 (10.4)
Student	347 (5.1)	4 709 (9.6)	5 056 (9.1)
Not reported	128 (1.9)	2 363 (4.8)	2 491 (4.5)
**Residence type**			
Cement building	2 930 (42.9)	36 174 (73.9)	39 104 (70.1)
Adobe	1 165 (17.0)	4 343 (8.9)	5 508 (9.9)
Tent	681 (10.0)	1 647 (3.4)	2 328 (4.2)
Log house	720 (10.5)	2 082 (4.3)	2 802 (5.0)
Tile-roofed house	839 (12.3)	2 974 (6.1)	3 813 (6.8)
Thatched cottage	237 (3.5)	879 (1.8)	1 116 (2.0)
Bamboo tower	227 (3.3)	667 (1.4)	894 (1.6)
Other	38 (0.6)	172 (0.4)	210 (0.4)
**Exposure risk**			
Spends majority of time in nature	5 643 (82.5)	27 883 (57.0)	33 526 (60.1)
Little to no time in nature	1 194 (17.5)	21 055 (43.0)	22 249 (39.9)

Note: Inconsistencies arise in some values due to rounding.

Of the 55 775 respondents, a total of 14 325 (25.7%) had basic knowledge and 41 450 (74.3%) did not. Respondents with different gender, age, marital status, education level, occupation, residence type and high exposure risk exhibited statistically significant differences in knowledge (*P*-value: < 0.01; Table 2). Among the participants, 14 295 (25.6%) demonstrated knowledge of preventive and first aid behaviour whereas 41 480 (74.4%) did not. There were significant differences in knowledge regarding prevention and first aid behaviour among respondents of different gender, age, marital status, educational level, occupation, residence type; and whether they had high exposure risk to snakes (*P*-value: < 0.01; Table 2).

**Table 2:** Comparative analysis of demographic factors influencing knowledge about snakebite among Chinese residents, 2022

Characteristic	No (%)						
	Knowledge about snakebites				Knowledge about prevention and first aid		
	Sufficient (*n* = 14 325)	Limited (*n* = 41 450)	Difference between groups, *P*		Sufficient (*n* = 14 295)	Limited (*n* = 41 480)	Difference between groups, *P*
**Gender**							
Male	7 908 (24.7)	24 109 (75.3)	< 0.01		7 104 (22.2)	24 913 (77.8)	< 0.01
Female	6 417 (27.0)	17 341 (73.0)			7 191 (30.3)	16 567 (69.7)	
**Age, years**							
< 18	352 (11.3)	2 769 (88.7)	< 0.01		404 (12.9)	2 717 (87.1)	< 0.01
18–40	9 345 (28.1)	23 952 (71.9)			9 065 (27.2)	24 232 (72.8)	
41–60	4 019 (29.1)	9 803 (70.9)			4 096 (29.6)	9 726 (70.4)	
> 60	609 (11.0)	4 926 (89.0)			730 (13.2)	4 805 (86.8)	
**Marital status**							
Married	9 759 (29.0)	23 927 (71.0)	< 0.01		9 828 (29.2)	23 858 (70.8)	< 0.01
Unmarried	3 756 (22.3)	13 092 (77.7)			3 659 (21.7)	13 189 (78.3)	
Divorced	630 (17.2)	3 043 (82.8)			596 (16.2)	3 077 (83.8)	
Widowed	180 (11.5)	1 388 (88.5)			212 (13.5)	1 356 (86.5)	
**Education level**							
No education	349 (9.1)	3 497 (90.9)	< 0.01		399 (10.4)	3 447 (89.6)	< 0.01
Primary school	788 (11.5)	6 043 (88.5)			818 (12.0)	6 013 (88.0)	
Middle school	1 996 (19.4)	8 311 (80.6)			1 884 (18.3)	8 423 (81.7)	
High school or technical secondary school	2 788 (22.4)	9 670 (77.6)			2 710 (21.8)	9 748 (78.2)	
Vocational institution	3 745 (34.1)	7 229 (65.9)			3 761 (34.3)	7 213 (65.7)	
Bachelor’s degree or above	4 659 (41.0)	6 700 (59.0)			4 723 (41.6)	6 636 (58.4)	
**Occupation**							
Farmer	1 667 (19.1)	7 071 (80.9)	< 0.01		1 650 (18.9)	7 088 (81.1)	< 0.01
Skilled labourer	1 733 (20.6)	6 680 (79.4)			1 592 (18.9)	6 821 (81.1)	
Service worker	1 529 (22.3)	5 313 (77.7)			1 381 (20.2)	5 461 (79.8)	
Self-employed	815 (22.8)	2 766 (77.2)			737 (20.6)	2 844 (79.4)	
Freelance	1 444 (30.6)	3 268 (69.4)			1 299 (27.4)	3 413 (72.4)	
Snake catcher or breeder	203 (24.2)	636 (75.8)			136 (16.2)	703 (83.8)	
Land and sea field operator	189 (32.0)	402 (68.0)			139 (23.5)	452 (76.5)	
Cadre employee	2 393 (27.5)	6 298 (72.5)			2 446 (28.1)	6 245 (71.9)	
Medical employee	2 872 (49.3)	2 949 (50.7)			3 334 (57.3)	2 487 (42.7)	
Student	974 (19.3)	4 082 (80.7)			1 015 (20.1)	4 041 (79.9)	
Not reported	506 (20.3)	1 985 (79.7)			566 (22.7)	1 925 (77.3)	
**Residence type**							
Cement building	11 448 (29.3)	27 656 (70.7)	< 0.01		11 872 (30.4)	27 232 (69.6)	< 0.01
Adobe	913 (16.6)	4 595 (83.4)			694 (12.6)	4814 (87.4)	
Tent	201 (8.6)	2 127 (91.4)			115 (4.9)	2 213(95.1)	
Log house	395 (14.1)	2 407 (85.9)			315 (11.2)	2 487 (88.8)	
Tile-roofed house	909 (23.8)	2 904 (76.2)			902 (23.7)	2 911 (76.3)	
Thatched cottage	124 (11.1)	992 (88.9)			93 (8.3)	1 023 (91.7)	
Bamboo tower	216 (24.2)	678 (75.8)			169 (18.9)	725 (81.1)	
Other	119 (56.7)	91 (43.3)			135 (64.3)	75 (35.7)	
**Exposure risk**							
Little to no time in nature	5 943 (26.7)	16 306 (73.3)	< 0.01		6 829 (30.7)	15 420 (69.3)	< 0.01
Spends most of the time in nature	8 382 (25.0)	25 144 (75.0)			7 466 (22.3)	26 060 (77.7)	
**Experienced a snakebite**							
Yes	2 021 (29.6)	4 816 (70.4)	< 0.01		1 499 (21.9)	5 338 (78.1)	< 0.01
No	12 304 (25.1)	36 634 (74.9)			12 796 (26.1)	36 142(73.9)	

Notes: We used a *χ*^2^-test to calculate statistical significance between the different groups. Inconsistencies arise in some final values due to rounding.

Table 3 presents data on antivenom knowledge and first aid practices. For example, 55.5% (30 933/55 775) of respondents were aware of the existence of antivenom. We also identified numerous incorrect first aid practices among respondents, for example: rinsing the wound with liquids such as soapy water, alcohol, vinegar, or soy sauce; smearing the wound with toothpaste; cauterizing the wound; cupping or syringing the wound; tourniquet above the wound; or emergency treatments with folk remedies, among others. 

**Table 3:** Highlights from questions on antivenom knowledge and first aid practices, China, 2022

Question	No. of respondents (%) (*n* = 55 775)
**Do you know about antivenom?**	
Yes	30 933 (55.5)
No	24 842 (44.5)
**Which of the following is a method for dealing with the wound of a snakebite?**	
Rinse the wound with soapy water or alcohol for at least 15 minutes^a^	27 335 (49.0)
Vinegar or soy sauce rinse for 15 minutes^a^	14 684 (26.3)
Smear the wound with toothpaste^a^	14 335 (25.7)
Leave the wound in soapy water for at least 15 minutes^a^	16 033 (28.7)
Do not know	14 736 (26.4)
**Which of the following is a first aid practice after a snakebite?**	
First, sit down and reduce movements to avoid accelerating blood circulation and rapidly spreading the venom	23 983 (43.0)
Search for the offending snake and kill or capture the snake^a^	11 814 (21.2)
Search for help (call an ambulance, passers-by, friends)	26 948 (48.3)
Identify the type of venomous snake so it can be treated appropriately	17 857 (32.0)
Wash the wound with soapy water, cold water, spring water, and try to wash the venom out of the bite^a^	17 848 (32.0)
Clean the wound with alcohol and iodophor^a^	11 017 (19.8)
Cauterize the wound 2–3 times^a^	5 905 (10.6)
Suck out the venom with Chinese cupping or a syringe	7 971 (14.3)
Using a bandage or rope, ligation is performed near the centre of the bite^a^	11 028 (19.8)
Use a branch or splint to immobilize the injured limb	8 411 (15.1)
Emergency treatments with relevant folk remedies	4 265 (7.6)
Go to a medical facility	15 071 (27.0)
Do not know	8 686 (15.6)

^a^ Indicates incorrect first aid practice.

Note: These questions are based on Chinese expert consensus on snakebite^8^ and related studies in India,^14^ as well as the design after group discussions and expert interviews.

### Multivariate analyses

#### Basic knowledge

Our multifactor logistic regression model indicates that individuals aged between 18 and 60 years had better basic knowledge about snakebite than those individuals younger than 18 years. Married individuals had more knowledge than all other groups (Table 4). Compared to individuals without any formal education, individuals partially educated at a primary level were found to have better basic understanding of information about snakes and snakebite prevention and first aid. 

**Table 4:** Multifactor logistic regression analysis on determinants of basic knowledge and prevention and first aid knowledge about snakes and snakebite, China, 2022

Variable	OR (95% CI)	
	Basic knowledge	Prevention and first aid knowledge
**Gender**		
Male	Reference	Reference
Female	1.000 (0.959–1.042)	1.305 (1.251–1.360)
**Age, years**		
< 18	Reference	Reference
18–40	1.511 (1.330–1.717)	1.231 (1.087–1.393)
41–60	1.744 (1.527–1.993)	1.479 (1.300–1.684)
> 60	0.827 (0.707–0.967)	0.788 (0.678–0.915)
**Marital status**		
Married	Reference	Reference
Unmarried	0.667 (0.632–0.704)	0.658 (0.622–0.695)
Divorced	0.657 (0.597–0.723)	0.651 (0.589–0.719)
Widowed	0.508 (0.431–0.600)	0.622 (0.532–0.728)
**Education level**		
No education	Reference	Reference
Primary school	1.333 (1.163–1.528)	1.263 (1.107–1.441)
Middle school	2.397 (2.111–2.722)	2.006 (1.773–2.270)
High school or technical secondary school	2.884 (2.537–3.279)	2.472 (2.182–2.800)
Vocational institution	4.717 (4.142–5.370)	3.970 (3.498–4.506)
Bachelor’s degree or above	5.903 (5.177–6.731)	4.717 (4.150–5.362)
**Occupation**		
Farmer	Reference	Reference
Skilled labourer	0.805 (0.741–0.874)	0.735 (0.676–0.799)
Service worker	0.773 (0.708–0.844)	0.680 (0.621–0.744)
Self-employed	0.741 (0.668–0.822)	0.623 (0.561–0.693)
Freelance	1.028 (0.938–1.127)	0.833 (0.758–0.915)
Snake catcher or breeder	1.424 (1.189–1.707)	0.940 (0.765–1.155)
Land and sea field operator	1.830 (1.505–2.226)	1.352 (1.093–1.672)
Cadre employee	0.837 (0.770–0.910)	0.842 (0.774–0.917)
Medical employee	2.098 (1.922–2.291)	2.800 (2.562–3.061)
Student	0.830 (0.745–0.926)	0.819 (0.734–0.914)
Not mentioned	0.917 (0.811–1.036)	0.871 (0.773–0.982)
**Residence type**		
Cement building	Reference	Reference
Adobe	0.580 (0.535–0.627)	0.443 (0.406–0.483)
Tent	0.283 (0.243–0.329)	0.163 (0.134–0.198)
Log house	0.465 (0.415–0.522)	0.365 (0.322–0.413)
Tile-roofed house	0.931 (0.857–1.011)	0.948 (0.872–1.030)
Thatched cottage	0.367 (0.302–0.447)	0.264 (0.212–0.330)
Bamboo tower	0.964 (0.818–1.136)	0.681 (0.570–0.814)
Other	3.721 (2.752–5.030)	4.922 (3.602–6.727)
**High exposure risk **		
Little to no time in nature	Reference	Reference
Spends most of the time in nature	1.400 (1.339–1.464)	1.019 (0.974–1.066)

CI: confidence interval; OR: odds ratio.

Snake catchers or breeders, and land and sea field operators had higher odds of knowing about snakes and snakebite than farmers: odds ratio (OR): 1.424 (95% CI: 1.189–1.707) and OR: 1.830 (95% CI: 1.505–2.226), respectively. Those who spend more time in the nature had better basic knowledge than those who did not: OR: 1.400 (95% CI: 1.339–1.464; Table 4).

#### Prevention and first aid 

Our multifactor logistic regression model shows that females (OR: 1.305; 95% CI: 1.251–1.360) score higher in prevention and first aid practices than males. The cohort between 18–60 years old exhibited a broader understanding than their counterparts younger than 18 years. Being married and having some form of higher education suggested one had better preventive and first aid-related practices than uneducated and not married respondents (Table 4).

## Discussion

Our results show that only one quarter of respondents had basic knowledge about snakes and snakebite, which is lower than reported results (35%) from a study of 100 respondents in India.^11^ Furthermore, only one quarter of residents had basic knowledge of prevention and first aid practices for snakebite, which is lower than reported results for India (64.2% of 227 respondents) and Myanmar (39% of 4276 respondents).^12^^,^^15^ Another recent study from China also highlights the need to improve residents' knowledge of snakebite first aid and prevention.^9^

In 2019, WHO released *Snakebite envenoming: a strategy for prevention and control*, a document which established numerous prevention and control strategies designed to halve mortality and disability rates related to snakebite by 2030.^16^ For example, antivenom is considered to be the most effective treatment for snakebite.^17^^–^^19^ The results of a pilot survey in India showed that only 21.0% of the 100 participants surveyed knew the type of antivenoms used in India;^11^ compared to 87.6% (148/176) of participants in Sri Lanka, who were aware of the existence of antivenom and which hospitals stocked it in case of emergency.^20^ This result may be due to several factors, such as early research programmes in 1965 to study snakebite in Sri Lanka;^21^ the high level of education, and residents' good understanding of snakebite.^20^ In China, we found that only 55.5% of respondents were aware of the existence of antivenom; let alone where to access it in an emergency.

Prevention and first aid are essential to reduce the risk of being bitten, as well as timely management of any bites to reduce complications and save lives.^22^^–^^24^ Using the wrong first aid method to treat a snakebite not only costs valuable time but also increases the risk of complications or death for the patient.^25^^,^^26^ However, in our survey results, basic knowledge of preventive and first aid practices is low. The results may be due to the fact that individuals do not give enough attention to snakebite-related illness or injury, believing that the possibility of being bitten by poisonous snakes is minimal and will not lead to serious consequences;^16^ or these results may be due to limited effective health guidance. For example, evidence for strapping or bandaging varies. One study in Nigeria showed that strapping may cause severe local swelling, negatively affecting the outcome of later treatments.^25^ In cases of extreme neurotoxicity, evidence suggests that strapping or bandaging is a recommended first aid method.^8^^,^^27^ From this we can conclude that not including snakebite envenoming in public health programmes means there is limited knowledge of snakebite prevention and first aid practices among the general population, which is consistent with the studies conducted in India, among others.^12^^,^^28^^–^^31^ Therefore, teaching individuals correct prevention and first aid practices for different types of snakebite prevalent in their local area is necessary to reduce human morbidity and mortality from snakebite envenoming.

Our analysis suggests that a higher proportion of women have preventive and first aid knowledge than men. This outcome may be because women have a stronger fear of snakes than men,^32^ so they are more likely to learn about and adopt snakebite prevention and first aid practices. Respondents older than 60 years had the lowest scores. In China, the belief that snakes selectively bite only those who are morally corrupt, coupled with the idea that various snake species symbolize either positive or negative omens, is a widely held superstition. This misconception often overlooks the medical significance of snakebite. Reports suggest that such beliefs are also prevalent in India.^31^^,^^33^^,^^34^

Married residents tend to be better informed and exhibit more appropriate snakebite prevention and first aid practices. In contrast, those who are unmarried, divorced or widowed may not have a spouse to communicate such knowledge, potentially resulting in less access to information compared to their married counterparts.^35^ Additionally, we found that the higher an individual's level of education, the more proficient they are in their knowledge and first aid practices about snakebite. This outcome may be, for example, because educated individuals have access to a wider range of information sources, such as magazines, social media platforms and educational websites, coupled with a greater capacity to assimilate knowledge and information about snakebite.

In India, rural residents with limited literacy obtain snakebite knowledge primarily from their immediate social circle and environment.^11^ Yet, this practice is problematic if the social circle possesses limited knowledge as well.^36^ For risk occupations, like snake catchers or breeders, there is foundational knowledge about snakebite due to the nature of their work. However, our study found that this career advantage does not substantially translate to better prevention or first aid knowledge, potentially due to the small sample size of such professionals. Our study has other limitations. The snakebite knowledge and behaviour questions could have been inadequate and lacked applicability across diverse geographic populations, misrepresenting participants' actual understanding of the concepts. Second, as this was a cross-sectional study, cause and effect could not be determined and recall bias could not be avoided.

One advantage of our study is that it reports on multiple demographic and social factors influencing snakebite knowledge and behaviour across 12 provinces and cities in China. We also supplemented the data obtained from face-to-face surveys with online surveys directed to WeChat and QQ users across the country. Given China's large population size (> 1 billion), drawing from national data can enhance statistical power. 

To improve knowledge, educational sessions on snakebite, including lectures and video documentaries could be provided in public schools in high-risk areas, as done in a study from India. The sessions in this study were accompanied by interactive question and answer sessions and distribution of informative leaflets, with students being encouraged to share the information with their families, friends and neighbours.^14^ such approaches are deemed effective because schools are pivotal for health education activities,^37^ and new media channels are an efficient means to spread health education programmes more broadly.^38^^,^^39^ Another Indian study found that over 85.0% of 413 participants demonstrated high retention of information even 12 months after training.^14^ Our study indicates that farmers with low education levels have poor knowledge of snakebite; prior studies suggest that digital media platforms and situational simulations could significantly enhance learning in these demographics.^40^

Both WHO and research from India and Nepal highlight the critical role of community health workers, educators and local officials in raising awareness about snakebite via radio, leaflets, posters and educational sessions.^14^^,^^16^^,^^41^ The development of mobile applications to identify high-risk areas, the listing of local health-care facilities capable of treating snakebite and offering remote video guidance for treatment have all been shown to improve treatment rates.^41^^–^^44^ Consequently, we suggest that relevant departments should increase funding for such research projects, and support scientific research and community intervention programmes tailored to the population's needs.

In conclusion, enhancing the education and dissemination of knowledge regarding snakebite is crucial, particularly for at-risk populations, through various media and community-based interventions.
